# Perspective change: Students as instructors – a report of experiences by nursing and medical students in the role of peer tutors

**DOI:** 10.3205/zma001840

**Published:** 2026-04-15

**Authors:** Patrick Frei, Tim Fischer, Jan-Ole Heering, Sarah Gutmann

**Affiliations:** 1 ETH Zürich, Bachelor of Medicine, Zürich, Switzerland; 2 Berner Bildungszentrum Pflege, Bern, Switzerland

**Keywords:** peer tutoring, nursing students, medical students, practical skills

## Abstract

**Introduction:**

Peer tutoring is a well-established approach to supporting learning that encourages knowledge exchange and social interaction among students. In this setting, more experienced students take on the role of tutors and assist their peers in developing both theoretical understanding and practical skills. At the Bern College of Nursing (BZ Pflege) and the Swiss Federal Institute of Technology Zurich (ETH Zurich), this approach is specifically applied to teaching the insertion of peripheral venous catheters (PVCs). In this article, four peer tutors share their reflections on this experience, highlighting both the challenges and the benefits of this teaching method.

**Process description:**

The preparation of peer tutors is structured through a comprehensive training program, which includes theoretical online preparation, practical skills training, and didactic instruction. During the teaching sessions, tutors lead small groups, demonstrate peripheral venous catheter (PVC) insertion according to Peyton’s Four-Step Approach, and supervise students as they practice. Insights were gathered through peer-to-peer discussions among the tutors, feedback from course participants, and self-reflection.

**Results:**

Peer tutoring benefits not only the learners but also the tutors themselves, enhancing their professional, didactic, and social competencies. Tutors improve their communication and leadership skills, develop problem-solving strategies, and strengthen their decision-making abilities. Supervising small groups creates a supportive learning environment and allows for individualized adaptation of instruction to meet students’ needs.

**Conclusion:**

Peer tutoring is a valuable complement to traditional teaching methods. Students benefit from practical, hands-on instruction and direct feedback, while tutors acquire important competencies for their professional practice. Therefore, peer tutoring should be further promoted and integrated into education.

## Zusammenfassung

**Einleitung:**

Peer-Tutoring ist eine bewährte Methode zur Lernförderung, die den Wissensaustausch und soziale Interaktion unter Studierenden stärkt. Erfahrenere Studierende übernehmen die Rolle der Lehrenden und unterstützen ihre Peers in theoretischen und praktischen Fertigkeiten. Am Berner Bildungszentrum Pflege (BZ-Pflege) und der ETH Zürich wird das Projekt gezielt zur Vermittlung des peripheren Venenkatheter (PVK) Legens eingesetzt. In diesem Beitrag reflektieren vier Peer-Tutorierende ihre Erfahrungen und beleuchten Herausforderungen sowie Vorteile dieser Lehrmethode.

**Prozessbeschreibung:**

Die Vorbereitung erfolgt durch ein strukturiertes Schulungsprogramm, mit theoretischer Online-Vorbereitung, praktischer Technikschulung und didaktischem Training. Im Unterricht leiten die Tutorierenden Kleingruppen an, demonstrieren die PVK-Anlage nach der Vier-Schritt-Methode nach Peyton und begleiten die Studierenden beim Üben. Erkenntnisse wurden durch Austausch unter den Tutorierenden, Feedback der Kursteilnehmenden und Selbstreflexion gewonnen.

**Ergebnisse:**

Peer-Tutoring kommt nicht nur den Lernenden zugute, sondern fördert auch fachliche, didaktische und soziale Kompetenzen der Tutorierenden. Sie verbessern ihre Kommunikations- und Führungskompetenzen, entwickeln Problemlösungsstrategien und stärken ihre Entscheidungsfähigkeit. Die Betreuung in kleinen Gruppen schafft eine lernförderliche Atmosphäre und ermöglicht individuelle Anpassung des Unterrichts.

**Schlussfolgerung:**

Peer-Tutoring ist eine wertvolle Ergänzung traditioneller Lehrmethoden. Studierende profitieren von praxisnahem Unterricht und direktem Feedback, Tutorierende erwerben wichtige Kompetenzen für ihre berufliche Tätigkeit. Daher sollte Peer-Tutoring weiter gefördert und in die Ausbildung integriert werden.

## 1. Introduction

Peer tutoring is a method of learning support that promotes knowledge exchange and social interaction among learners by having more experienced students assist less experienced ones in better understanding and applying content [1]. Peer tutors are themselves learners or students who teach, guide, and support their peers’ learning progress by sharing their knowledge and skills. By teaching, peer tutors deepen their own knowledge and competencies in the respective area [2]. In this collaborative learning, learners of equal standing are actively involved in the teaching process. Instead of the instructors, the students take over the role of teachers and convey both theoretical and practical skills to their peers [2].

Peer tutoring is already being used at various colleges and universities. The advantages of peer tutoring include, among others, the promotion of personal responsibility, independent learning, and the ability to reflect. In addition, it enables a trusting learning environment through horizontal communication between students [3].

The insertion of a peripheral venous catheter is well suited for peer tutoring, as it is a common and practice-oriented procedure in clinical routine. In a protected learning environment, students can guide each other practically, provide feedback, and thereby strengthen both professional and communication competencies. The clear structure and the integration of theory and practice promote effective, collegial learning on an equal footing [4]. Peer tutors are deployed in the third semester during practical instruction for PVC insertion. This takes place in the facilities of the Learning, Training, and Transfer (LTT) centre of a nursing school – a concept that has been established for years and proven effective in practice [5]. The collaboration between the Bern College of Nursing (BZ-Pflege), a higher vocational nursing school in Bern, and the Swiss Federal Institute of Technology Zurich (ETH Zurich) with the bachelor’s degree in medicine forms the basis of the course, linking nursing and medical education with didactic innovation.

The setting of the course is designed to be practice-oriented: while lecturers from BZ Pflege teach the theoretical part, peer tutors from medicine and nursing conduct the practical training. In this way, theoretical knowledge is directly linked to clinical skills.

At ETH Zurich, a similar project with peer tutors is conducted within the bachelor’s program in Human Medicine. While some literature has already been published on the concept and application of peer tutoring, the voices of the peer tutors themselves remain insufficiently represented. Therefore, the question of how peer tutors perceive their role from the perspective of their own experiences remains largely unanswered.

## 2. Process description

In this article, the four authors describe their experiences as peer tutors and reflect on them from their own perspective.

### 2.1. Training of the peer tutors

To become peer tutors at BZ Pflege and at the Skills Lab of ETH Zurich, we – three medical students and one nursing student – responded to a call for applications addressed to students from the fourth semester onwards of their respective programs. Applicants who met the required profile then completed a structured preparatory program that included the following elements: an online study of the theoretical foundations regarding procedures, indications and contraindications, and material knowledge for the insertion of a peripheral venous catheter (PVC), implemented in the form of a flipped classroom. In a subsequent on-site technical training, the theoretical foundations were deepened, and the practical skills were practiced on models. This technical training standardized the procedure to ensure that the teaching aligns with current standards, thereby guaranteeing quality and safety. The technical training provided us, as future peer tutors, with confidence and assurance. Finally, in a four-hour didactic training session, teaching styles, communication strategies, and classroom management were addressed. This part focused on communication and behaviour during instruction, which prepared us peer tutors for conducting the classes.

### 2.2. Implementation of the teaching sessions

During the teaching sessions, we as peer tutors supervised groups of three to four students. Due to the preceding theoretical instruction, the students already possessed prior knowledge, which was further deepened during the practical training of peripheral venous catheter (PVC) insertion. The teaching followed Peyton’s four-step approach [6]. This structured method facilitates the understanding of complex procedures and promotes a safe and systematic application of the technique. In addition to activating the students’ prior knowledge and introducing the materials, the focus was placed on the practical insertion of the PVC. As a supplement, we demonstrated blood collection and the flushing of the vein with a sodium chloride solution using a model, before the students were able to practice the insertion themselves on venous arms and cuffs. Once they felt sufficiently confident, they were allowed to practice voluntarily on each other – however, without performing blood collection or venous flushing. We conducted the entire practical part independently, while a nursing professional was available for support in case of questions or complications. In the event of an incident, we were covered by institutional insurance.

### 2.3. Different teaching programs

In the bachelor’s program in Human Medicine at ETH Zurich, peer tutoring is integrated into the Skills Lab and includes voluntary tutorials for learning practical skills. The selection of tutorials offered – including PVC insertion, blood sampling, injections, ultrasound, suturing, wound care, and digital training in traumatology – is designed to prepare students for their future medical practice.

At BZ Pflege, peer tutoring is implemented in a similar way: practical training in the facilities of the Learning, Training, and Transfer (LTT) centre is an integral part of the curriculum and is supported by peer tutors in various areas.

### 2.4. Summary of experiences

At the beginning, we formulated three guiding questions to filter relevant aspects and consolidate insights:

- What motivates us to engage in teaching?
- Which conditions are necessary for successful instruction?
- Which strategies did we apply to improve our teaching?

Initially, each of us wrote an individual report of experiences. For the further analysis of our teaching activities, mutual exchange was central; therefore, we subsequently combined our experiences and derived shared insights.

## 3. Results

### 3.1. Sharing knowledge, multiplying success

The teaching method of peer tutoring not only provides students with an optimal learning platform but also gives us, the peer tutors, the opportunity to develop ourselves and to learn how to teach. Unlike nursing education, which is a dual program, the three of us medical students in the third year of study had little experience in PVC insertion when we attended the tutor training at BZ Pflege.

At our first deployment as peer tutors, our prior experience was very limited. Therefore, collaboration with experienced peer tutors was extremely helpful, as we were initially hesitant and relied on support. Through peer tutoring, we were able to expand our teaching competencies from the very first session. By demonstrating the procedure step by step, we consolidated our own skills and abilities. Regular teaching sessions allowed us to review knowledge, gain more routine and increase self-confidence in our practice. Our own experience and familiarity with the procedures significantly enhanced the quality of instruction. At the beginning, we observed that uncertainties and mistakes during demonstrations were often transferred to the students. The resulting corrections proved to be very time-consuming and challenging, making it essential to avoid inaccuracies when demonstrating. Moreover, the students’ questions constantly prompted us to reflect on and reconsider our actions and statements. These experiences were consistently motivating, and teaching brought us great joy. Through repeated delivery of teaching sessions, we continuously expand our professional and didactic competencies and gain confidence. This increasingly allows us to create a supportive and effective learning environment, apply insights in practice, and share knowledge with enthusiasm.

### 3.2. Expanding competencies for life

Through our work as peer tutors, we are able to gain important experiences and skills for life. These include intra- and interprofessional collaboration, as well as time management and coordination of a teaching session. These skills are essential for successful instruction. A challenge in intra- and interprofessional collaboration is that different didactic methods and levels of experience must be combined. However, this also offers opportunities for new ideas, methods, and engaging discussions. Peer tutors benefit from one another, can exchange ideas, and make the teaching sessions more dynamic. Good communication before the course begins is essential to prevent misunderstandings during instruction. Effective time management and coordination during the course reduce stress and promote a relaxed learning environment. Designing the lessons ourselves encourages us to utilize our own competencies and apply resources appropriately. Optimizing these aspects creates a positive learning atmosphere, contributing to a safe learning environment for the participants. In addition, our sense of responsibility and our ability to make independent decisions within a protected framework are strengthened. Our skills in knowledge transfer and communication can be practiced and improved through teaching. By independently conducting the lessons, we take responsibility for our own thinking and actions. This serves as excellent preparation for our future professional life. As qualified nurses and physicians, we will be responsible daily for important decisions and interactions with various professional groups, patients, and relatives. Moreover, we learn to reflect on and justify our actions, which strengthens our argumentation skills in future clinical practice. At times, we need to correct students to avoid potentially risky situations. On the other hand, we must be able to justify when an intervention that might unsettle students is not necessary. This fosters initiative and leadership skills. Additionally, we are encouraged to engage in problem-solving thinking, which helps us address issues effectively and find timely, efficient, and satisfactory solutions in our later professional work. Through peer tutoring, not only are competencies for successful teaching promoted, but we are also comprehensively prepared for our future clinical practice. This perspective has been examined in a study, which confirmed that peer tutoring improves the skills and knowledge of peer tutors for later professional practice [7].

### 3.3. Relationship with students and faculty

Our experiences, as well as positive feedback, show that students attend the PVC courses with enthusiasm when taught by us peer tutors. Although some course participants had never held a PVC before, by the end of the course all students had internalized the procedure and successfully inserted a PVC. This not only excites the students but also motivates us as peer tutors.

The opportunity for medical tutors to train nursing students together with nursing tutors further strengthens the relationship between future physicians and nurses. Since we are all still students ourselves, we automatically interact on an equal footing. This creates a pleasant, non-judgmental learning environment that allows for mistakes.

In addition to differences in study programs, group dynamics and skill levels always vary considerably. Therefore, the teaching must be spontaneously adapted to the current needs. For example, challenges can be incorporated for students with prior experience to stimulate and advance their learning, or lessons can be simplified with more detailed explanations for students with little experience. Peer tutoring enables close supervision and provides space to clarify open questions.

An important aspect of the peer tutor role is recognizing that some questions exceed one’s own knowledge. Initially, this was difficult to accept, but we continuously learn from the students’ questions. Feedback indicates that participants are motivated in class and value our time – an important affirmation of our role and the concept.

The larger number of tutors allows for more individualized supervision, which is particularly appreciated during practical learning sessions. This improves teaching quality and is recognized by instructors as valuable support. At the same time, we learn to appreciate the instructors. Being part of the lesson planning allows us to experience firsthand the considerable effort involved in preparing, conducting, and following up on the PVC course. This provides us with a new perspective on our studies and increases our appreciation for being able to attend such courses and lectures.

### 3.4. Continuous improvement of teaching sessions

As peer tutors, we constantly ask ourselves how we can improve both ourselves and the teaching. This is best achieved through student feedback and self-reflection, with self-reflection playing the more important role in our experience. It provides us with the opportunity to consider all aspects of the instruction and improve them. Therefore, it is our most important tool for training students at a high level. In addition, direct feedback from students helps us correct mistakes or maintain successful elements. Students have generally provided very positive feedback, which strengthens our confidence in teaching and confirms the effectiveness of this instructional model. However, especially at the beginning, it is important to listen to students’ needs and allow them to actively shape the course. Accordingly, we always share our new insights with the other peer tutors and instructors, discussing the implementation of feedback and reflections together. This gives us the opportunity to improve our competencies and adapt the teaching to a wide range of expectations and needs.

## 4. Discussion

### 4.1. Teaching suitable for beginners

One of the most important advantages for us as peer tutors is that we are still students ourselves and were recently in the same position as the learners. This allows us to understand their level of knowledge well and to tailor the teaching specifically to their needs. The literature confirms that peer tutors are particularly well able to address the needs of learners [8].

We, as peer tutors, have only recently learned the skills for PVC insertion ourselves and remember the initial difficulties very well. This enables us to provide students with beginner-friendly and practice-oriented tips that helped us when we were learning. This proximity to our own learning experience promotes a supportive learning environment, as also demonstrated by Yu et al. in their meta-analysis [9]. Furthermore, our authority as peer tutors is often perceived as less hierarchical, which facilitates students’ active participation and their willingness to ask questions. This effect is highlighted by Burgess et al., who show that flat hierarchies promote learner engagement and motivation [10].

Since the PVC course represents the first opportunity for many students to perform a PVC insertion, practicing on one another constitutes a significant stress situation. This stress is often caused by the fear of making mistakes. The fear is further intensified by the fact that an error can cause pain or minor bleeding. To counteract this first factor in particular, we aim to create an environment that allows mistakes, which we have been able to implement successfully for the reasons mentioned above.

Another advantage of peer tutoring is the possibility to form very small learning groups of a maximum of four students. This allows, firstly, much more individualized attention to the needs and questions of each student. Secondly, it enables greater practice and internalization of procedures when learning practical skills. Ultimately, this leads to greater learning success and increased confidence among the students.

### 4.2. Promotion of teaching competencies

In addition to the numerous benefits that peer tutoring offers students, it also contributes to the development of teaching competencies in young, inexperienced adults. Through teaching, various important skills for future professional life are acquired and fostered. Moreover, motivated young adults are given the opportunity to begin teaching early and to lay the foundation for potential future teaching activities within a protected environment.

Important skills that can be learned through tutoring include social and instructional competencies. Social competencies include, for example, intra- and interprofessional collaboration, responding to students’ needs and questions, empathy towards students, and communication skills during instruction. Instructional competencies encompass abilities such as time management, lesson planning, and the design and demonstration of teaching content. These skills are particularly essential in medical professions, as knowledge transfer will constitute a major part of daily professional practice.

## 5. Conclusion

Peer tutoring is an effective teaching method that provides substantial benefits for both tutors and students in health education. Through independent instruction and intensive engagement with practical skills such as peripheral venous catheter (PVC) insertion, peer tutors expand their professional, didactic, and social competencies. Students benefit from individualized supervision and a safe learning environment, which reduces fear of mistakes and promotes active participation. Furthermore, peer tutoring prepares participants for the challenges of professional practice by strengthening responsibility, communication skills, and problem-solving abilities. Thus, peer tutoring represents a valuable complement to traditional teaching methods and should be further promoted and expanded in health education.

## Authors’ ORCIDs

- Tim Fischer: [0009-0002-9357-8831]
- Sarah Gutmann: [0009-0004-5867-3854]

## Acknowledgements

We would like to thank Dr. Claudia Schlegel, MME, and Mirdita Useini, MME, from ETH Zurich for their support in the implementation of the peer tutoring sessions.

## Competing interests

The authors declare that they have no competing interests. 
